# Widespread distribution of *Aedes aegypti* larvae, a potential risk of arbovirus transmission in the Grand Lomé health region, Togo, West Africa

**DOI:** 10.1186/s13071-025-06835-7

**Published:** 2025-07-01

**Authors:** Kossivi I. Akagankou, Koffi M. Ahadji-Dabla, Daniel Romero-Alvarez, Juan-Carlos Navarro, Leonardo D. Ortega-López, Manuel Villanueva-Sarmiento, Komlan G. J. N’Tsoukpoe, Edoh Koffi, Yovo Kondo, Adjo A. Amekudi, Yawo Apetogbo, Audrey Lenhart, Guillaume K. Ketoh

**Affiliations:** 1 Laboratoire d’Ecologie Et d’Ecotoxicologie, Faculté des Sciences, Université de Lomé, 01 BP: 1515 Lomé1, Togo; 2 Emerging and Neglected Diseases, Research Group, Faculty of Health Sciences, School of Biomedical Sciences, Ecoepidemiology and Biodiversity, Universidad Internacional SEK (UISEK), Quito, Ecuador; 3 Instituto Nacional de Biodiversidad (INABIO), Quito, Ecuador; 4 Grupo de Investigación de Población y Ambiente, Universidad Regional Amazónica Ikiam, Tena, Ecuador; 5 Independent, Los Olivos, Lima, Peru; 6 Laboratoire d’Entomologie Fondamentale et Appliquée, Université Ouaga 1 Joseph Ki-Zerbo, Ouagadougou, Burkina Faso; 7 National Center for Emerging and Zoonotic Infectious Diseases, Division of Parasitic Diseases and Malaria/Entomology Branch, Centers for Disease Control and Prevention (CDC), Atlanta, GA 30329 USA

**Keywords:** *Aedes aegypti*, Ovitraps, Incidence, Land cover, Grand Lomé, Togo

## Abstract

**Background:**

Understanding the population dynamics and geographical range of *Aedes aegypti* is critically important for arbovirus vector surveillance and control. Little is known about the current distribution and seasonality of *Ae. aegypti* in Grand Lomé, Togo. We developed an investigation to determine whether *Ae. aegypti* was present across Lomé communes during a 1-year collection period.

**Methods:**

Mosquito ovitraps (*n* = 70) were deployed across the 13 communes in the Grand Lomé health region and were examined between May 2022 and April 2023. Generalized linear mixed models (GLMMs) were applied to investigate the relationship between larval collections and seasonality. The European Space Agency (ESA) WorldCover 10 m 2020 product was used to represent different land cover classes and to determine whether sites with higher larval numbers differed from sites with lower numbers.

**Results:**

A total of 52,768 *Ae. aegypti* larvae were collected across the 13 communes of Grand Lomé. The highest incidence of *Ae. aegypti* larvae was observed in the commune of Bè-Ouest (= 122.74 per 1000 population). Agoè-Nyivé was the commune with the lowest incidence over the entire study period. There was a statistically significant difference in *Ae. aegypti* larval counts between the rainy and dry seasons. Eight land-use classes were represented by the ESA 10 m product in Grand Lomé, with the built-up category being the most common. We found a significant relationship between larval abundance categories and land cover classes.

**Conclusions:**

This study shows that *Ae. aegypti* larvae can be found across all communes of the Grand Lomé region in both the rainy and dry seasons, especially in ovitraps surrounded by built-up land cover category. The results of this study could be useful in guiding disease vector surveillance and control efforts due to the potential imminent risk of upcoming dengue outbreaks.

**Graphical abstract:**

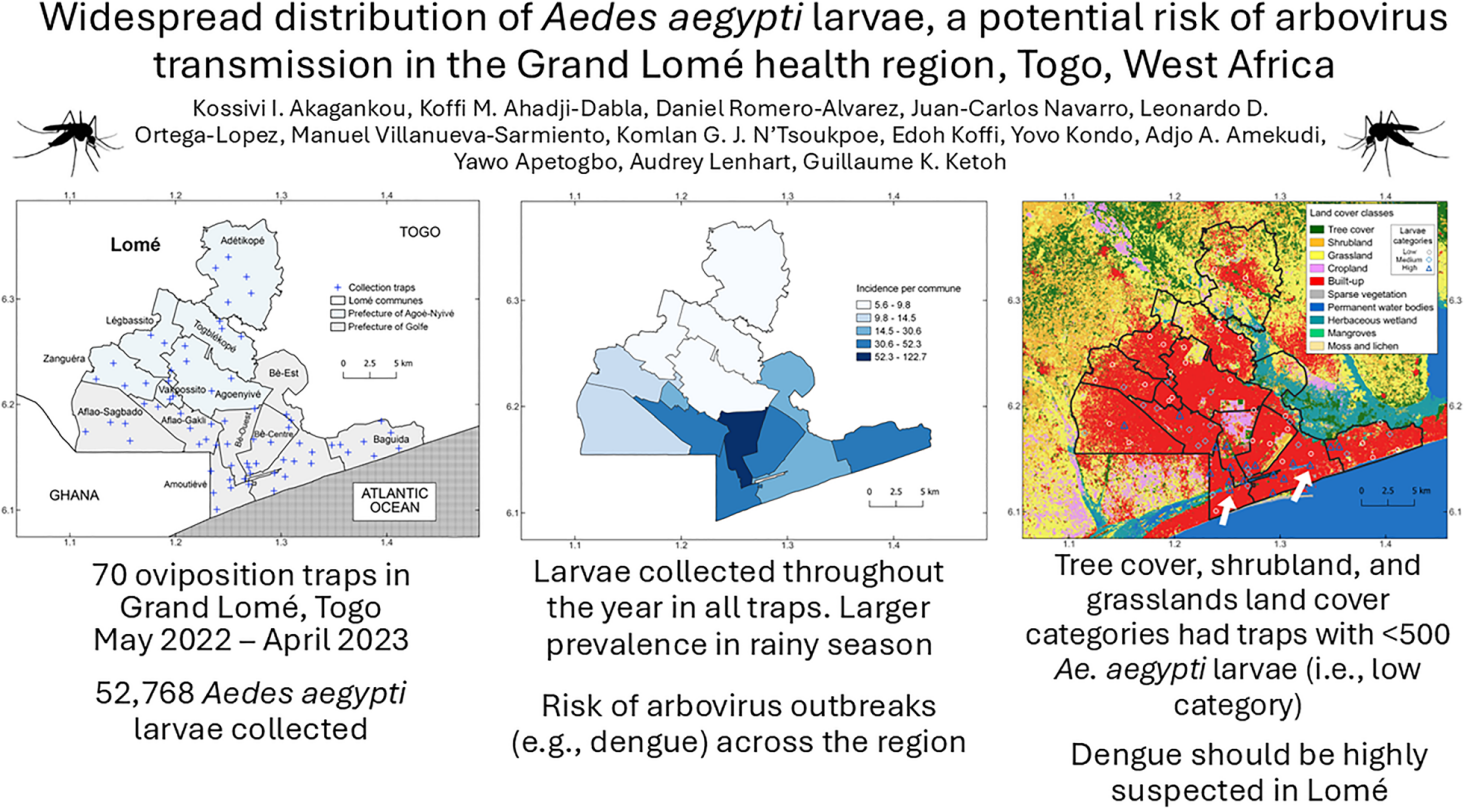

**Supplementary Information:**

The online version contains supplementary material available at 10.1186/s13071-025-06835-7.

## Background

*Aedes* (*Stegomyia*) *aegypti* (Diptera: Culicidae; Linnaeus, 1762) is an African native mosquito [1] that has spread and become established throughout the world, particularly in tropical and subtropical regions [2]. This mosquito can be found from sea level to altitudes as high as 2200 m in Colombia [3] and 2377 m in Africa [4]. *Aedes aegypti* was transported from Africa and introduced to the New World via the slave trade between the sixteenth and nineteenth centuries [5]. During World War II, *Ae. aegypti* was introduced into most Asian cities, then spreading from Southeast Asia to the Pacific region [5]. This mosquito is the main and most prolific vector involved in the transmission of neglected infectious diseases such as dengue, chikungunya, yellow fever, and Zika, all of them caused by arboviruses [6, 7].

Dengue in particular poses a serious and growing threat to public health worldwide [8]. Over the past 20 years, cases of dengue fever have risen dramatically worldwide, from 505,430 cases reported in 2000 to 5.2 million in 2019. Currently, it is estimated that around half the world's population is at risk of dengue [9]. Dengue is expanding rapidly, with 100 to 400 million new infections per year, and is now endemic to a growing number of tropical megacities [10, 11]. In 2023, more than 5 million cases and 5000 deaths were reported worldwide in over 80 countries across the six World Health Organization (WHO) regions [12]. Importantly, the number of dengue infections in the African region increased ninefold relative to 2019 [12], making it the most widely dispersed arboviral disease in West Africa [13]. This rapid increase in dengue cases is likely associated with the adaptation of *Ae. aegypti* to suitable anthropogenic environments such as abandoned tires [10]. Indeed, increasing urbanization could potentially alter the ecology of *Aedes* mosquitoes and increase the abundance of their breeding habitats [14]. In addition, urban areas are favorable for the rapid spread of arboviruses due to the presence of high human population density and complicit human migratory patterns [15].

In Togo, West Africa, little is known about the ecology and distribution of *Aedes* or other mosquitoes [16, 17]. The scant available works have focused on reporting the presence of the species in Togo. Recently, a study by Adjobi et al. [18] in Côte d’Ivoire showed high *Aedes* and arboviral risk indices in the capital city of Abidjan, and in Togo, a study conducted by Akagankou and collaborators in 2024 showed that rainfall exerted a slightly significant positive influence on the number of eggs, favoring high values for ovitrap positivity index and egg density per ovitrap, indicating a high risk of arbovirus transmission in the Grand Lomé health district. Less is known about the presence of dengue in Togo. A study in Lomé by Salou et al*.* (2017) reported that 17% of dengue cases were diagnosed with febrile syndromes [19]. Consequently, dengue fever is seldom suspected by health professionals in the country [20]. In 2023, for the first time, eight confirmed cases of dengue fever were officially reported [12]. Proximity and frequent movement of people between neighboring dengue-endemic countries such as Benin, Burkina Faso, and Ghana predispose Togo to an increased risk of dengue epidemics. Thus, understanding population dynamics and vector range expansion might be fundamental to anticipating dengue outbreaks in the country and building capacity for disease surveillance and vector control.

In this study, we sought to determine the presence of *Ae. aegypti* in Grand Lomé via an entomological collection implemented for approximately 1 year to understand the dynamics of *Ae. aegypti* larvae in the region during the rainy and dry seasons. We further used a landscape analysis to determine the characteristics of sites with the greatest abundance of *Ae. aegypti* mosquito larvae across Grand Lomé, suggesting communes to target surveillance efforts and strategies for vector control.

## Methods

### Study area

**Fig. 1:**
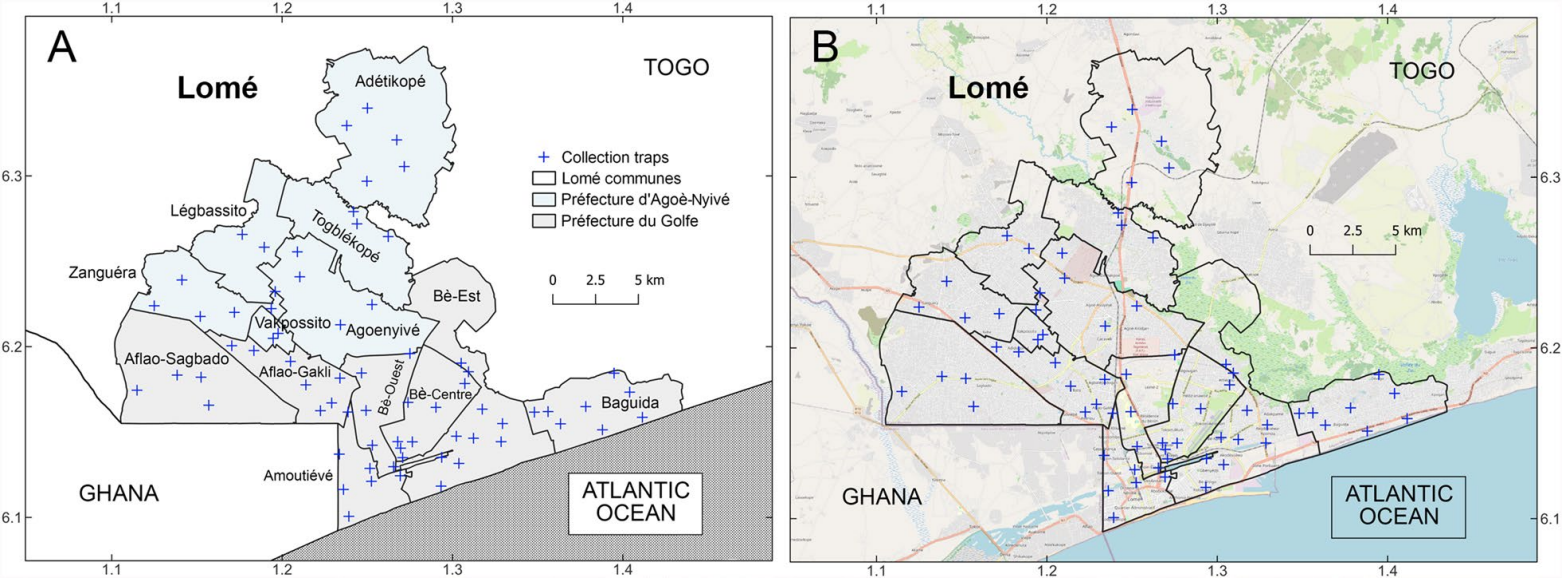
Distribution of ovitraps used for egg/larval collections across Grand Lomé, Togo. A total of 70 ovitraps were distributed across the 13 communes of Grand Lomé following a convenient and random approach aiming to maximize the probability of trap recovery across urban, peri-urban, and rural areas of Lomé in the prefectures of Golfe (gray) and Agoè-Nyivé (sky blue; **A**) between May 2022 and April 2023. Grand Lomé is the most densely populated city of the country, with few green areas (**B**, OpenStreetMap)

The study took place in the health region of Grand Lomé (6°7′54.998″N, 1°13′22.001″E), which is divided into two prefectures (Golfe and Agoè-Nyivé) and 13 communes: Bè-Est, Bè-Centre, Bè-Ouest, Amoutiévé, Aflao-Gakli, Baguida, and Aflao-Sagbado representing Golfe 1, 2, 3, 4, 5, 6, and 7, respectively; and Agoè-Nyivé, Légbassito, Vakpossito, Togblékopé, Sanguéra, and Adétikopé representing Agoè-Nyivé 1, 2, 3, 4, 5, and 6, respectively (Fig. 1). The health region of Grand Lomé covers an area of 425.6 km^2^ and is the most densely populated region of Togo, with a population of 2,188,376 in 2022, representing 27% of the national population [21]. Seasons, especially in Togo’s southern region, are roughly divided into the long rainy season (April–July), short dry season (August–September), short rainy season (October–November), and long dry season (December–March [22]). Except for the peri-urban communes (Golfe 6, 7, Agoè-Nyivé 2, 5, and 6; Fig. 1), where a few crops are still grown, agriculture in the Grand Lomé region is mainly limited to market gardening.

### Mosquito egg/larval collection

ata collection period was from May 2022 to April 2023.

A total of 70 oviposition traps were set up in households across the 13 Grand Lomé communes. According to the size and/or the population of each commune, a total of five or six ovitraps were supposed to be deployed in each commune, making a total of 70 ovitraps. However, adjustments were made to have a minimum of three and a maximum of 10 ovitraps per commune. Our entomological collection focuses only on larvae in this article.

The inclusion criteria for the deployment of ovitraps include the presence of used tires near human dwellings, insalubrity, and the presence of water reservoirs near dwellings [23]. The minimum distance between two ovitraps was set at approximately 500 m to minimize the collection of the same mosquito species and also to avoid dispersal from one commune to another [24]. Sites for trap deployment were selected conveniently and randomly in each commune but aiming to cover a variety of urban and non-urban contexts covering all the communes of the region. The traps consisted of 750 ml black cups filled two-thirds with tap water. Each cup was lined with brown paper to facilitate egg fixation. These traps were examined weekly between May 2022 and April 2023, with sampling occurring during the long rainy season and the short and long periods of the dry season.

Ovitraps were recovered periodically, and eggs and larvae were collected. Eggs were then reared to larvae in the insectary of the Laboratory of Ecology and Ecotoxicology of the University of Lomé. We used *Ae. aegypti* larvae collected directly in the field and reared in the laboratory as the unit of analysis and interpretation. All larvae were identified using the taxonomic keys of Edwards and Hopkins [25, 26]. Larvae were reared in the laboratory until adulthood, and we calculated the proportion of *Ae. aegypti* that reached adult stage. Adults were maintained in Eppendorf tubes containing silica gel according to their geographical origin, and then stored at −20 °C for subsequent analysis. Rearing conditions included temperature = 26 ± 2 °C, relative humidity (RH) = 85 ± 5%, and photoperiod = 12 L:12 D. In this study, we focused on the presence of *Ae. aegypti*; future reports will describe the presence of other relevant species collected.

### Estimating the potential for dengue emergence

As *Ae. aegypti* is the most important vector for dengue transmission, and due to recent official reports of dengue virus circulation in Lomé [12], we assumed that the number of *Ae. aegypti* larvae per commune divided by total commune population (i.e., incidence) could be interpreted as the risk of potential outbreaks of mosquito-borne diseases in Lomé. We estimated the incidence of larvae per 1000 human population in each of the Grand Lomé communes using 2022 population estimates [21]. Larval incidence was calculated for the entire study period, and independently for the rainy and dry seasons. Both *Ae. aegypti* larval incidence and total number per trap were visualized in maps using natural Jenks breaks [27]. Generalized linear mixed models (GLMMs) were applied to investigate the relationship between larval collections and seasonality [28]. Larval counts were set as the dependent variable and fitted with a Poisson distribution [29]. Seasonality with two categories (rainy and dry seasons) was set as a fixed effect, while collection site, prefecture, and commune were random effects. Model selection was performed using the likelihood ratio test (LRT) with the function *drop1* in R programming language [30]. The model formula was defined as follows:

$$Y_{ijkl} \sim {\text{Poisson}}\left( {\lambda_{{{\text{ijkl}}}} } \right)$$

$${\text{Log}}\left( {\lambda_{{{\text{ijkl}}}} } \right) \, = \beta_{0} + \beta_{{1}} *{\text{ Season}}_{{\text{i}}} + \beta_{{\text{j}}} + \beta_{{{\text{jk}}}} + \beta_{{{\text{jkl}}}} + \beta_{{{\text{ijkl}}}},$$

where λ_ijkl_ is the expected count of larvae, β_0_ is the intercept, β_1_ represents the effect of season, _j_ ~ *N* (0, σ^2^_prefecture_) is the prefecture-level random effect, _jk_ ~ *N* (0, σ^2^_commune_) is the commune-level random effect (within a prefecture), *w*_jkl_ ~ *N* (0, σ^2^_site_) is the site-level random effect (within a commune), and ε_ijkl_ is the residual error.

### Association between larval abundance and land cover

The European Space Agency (ESA) WorldCover 10 m 2020 product (https://worldcover2021.esa.int/) was used to define different land cover classes across Grand Lomé and to determine whether sites with larger numbers of larvae differed from sites with lower numbers [31]. The ESA WorldCover product is a high-resolution satellite product with 11 land categories derived from images captured by the Sentinel 1 and 2 satellites (https://worldcover2021.esa.int/). A buffer of 200 m was created around each collection site (*n* = 70), and the number of pixels belonging to any land cover category was counted (Table 4). To investigate whether larval abundance and land cover were related, the total number of *Ae. aegypti* larvae obtained at each collection site were classified into three categories: low (< 500 larvae), medium (500–1000 larvae), and high (> 1000 larvae). A contingency table was created with the number of pixels that corresponded to each land cover category, and we used a Chi-square test of independence to assess the association between larval abundance groups and land cover categories [32].

All statistical analyses were performed in the programming language R (version 4.3.2; https://www.R-project.org) through RStudio Pro (version 2023.12.1). GLMMs were carried out with the package *lme4* [33], and LRT and Chi-square tests were performed with the package *stats*.

## Results

A total of 70 ovitraps were deployed across the 13 communes of Grand Lomé between March 2022 and April 2023 (Fig. 1). The maximum number of ovitraps were set in the Bè-Est commune (*n* = 10; Table 1). The communes of Légbassito and Vakpossito had the lowest number of deployed traps (*n* = 3, each; Table 1). More than 75% of larvae reached adulthood (Table 1). All the mosquitoes identified as *Ae. aegypti* as larvae were also found to be *Ae. aegypti* in their adult stage (Pearson correlation, *Ae. aegypti* larvae–adult = 0.99).

**Table 1:** Overall counts of *Aedes aegypti* larvae and adults, and larval incidence in Grand Lomé

Communes	Commune capitals	Traps per commune	Human population	*Ae. aegypti* larvae	Incidence of *Ae. aegypti* larvae	Adult *Ae. aegypti*	Percentage adults
Agoè-Nyivé 1	Agoè-Nyivé	4	317,255	1788	5.63	1578	88.26
Agoè-Nyivé 2	Légbassito	3	128,164	1256	9.79	958	76.27
Agoè-Nyivé 3	Vakpossito	3	47,554	1453	30.55	1115	76.74
Agoè-Nyivé 4	Togblékopé	4	154,431	1253	8.11	1058	84.44
Agoè-Nyivé 5	Sanguéra	4	125,097	1818	14.53	1498	82.4
Agoè-Nyivé 6	Adétikopé	4	110,194	976	8.85	810	82.99
Golfe 1	Bè-Est	10	351,550	8525	24.24	7290	85.51
Golfe 2	Bè-Centre	6	136,153	4824	35.43	4053	84.02
Golfe 3	Bè-Ouest	5	52,769	6477	122.74	5387	83.17
Golfe 4	Amoutiévé	7	155,842	8153	52.31	6930	85
Golfe 5	Aflao-Gakli	7	169,993	6354	37.37	5310	83.57
Golfe 6	Baguida	8	181,561	6541	36.02	5325	81.41
Golfe 7	Aflao-Sagbado	5	257,813	3350	12.99	2890	86.27
Totals	Grand Lomé (total)	70	2,188,376	52,768	24.11	44,202	83.77

During 2022–2023, a total of 70 ovitraps were deployed across the 13 communes of Grand Lomé during the rainy and dry seasons. Eggs recovered were reared in the laboratory to count the number of larvae per commune and the number of *Ae. aegypti* reaching adulthood. Larval incidence was calculated using the human population of each commune in 2022 as denominator (see methods)

**Fig. 2:**
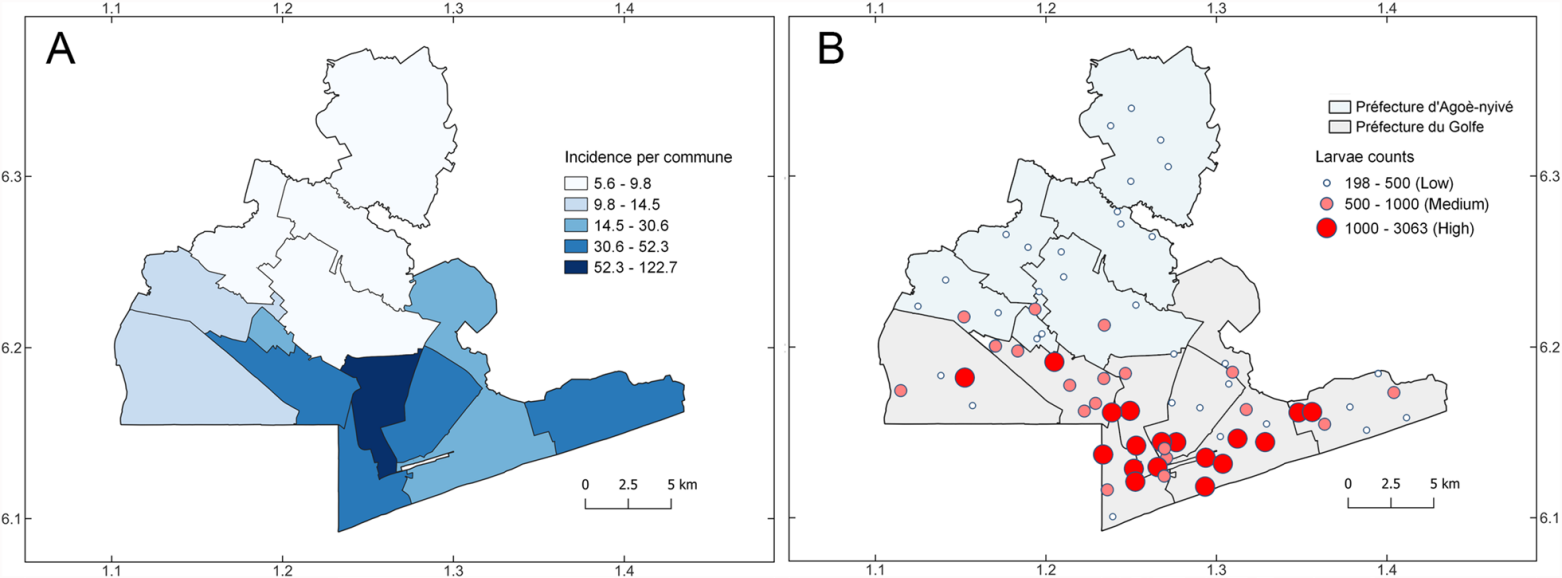
Overall*Aedes aegypti*larvae incidence per commune and counts of*Aedes aegypti*larvaeacross Grand Lomé, Togo. Eggs and larvae collected during 2022-2023 were reared in the laboratoryand the incidence of*Ae. aegypti* larvae per commune were calculated using total amounts of larvaedivided by total human population for 2022 times 1,000 (**A**). Counts of*Ae. aegypti* larvae per ovitrapwere categorized as low, medium or high to highlight patterns of larvae abundance across Lomécommunes (**B**)

In total, 52,768 *Ae. aegypti* larvae were collected in Grand Lomé across the 13 communes, throughout the entire study period, and from all 70 ovitraps. The highest incidence of *Ae. aegypti* larvae was detected in the Bè-Ouest commune (122.74 per 1000 population. This was followed in overall high incidence by the communes of Amoutiévé (52.32 per 1000 population) and Aflao-Gakli (37.38 per 1000 population; Table 1, Fig. 2A). Agoè-Nyivé was the commune with the lowest incidence across the entire study period (Fig. 2A). Traps with more than 1000 *Ae. aegypti* larvae were considered high and were mostly concentrated in the Golfe prefecture of Grand Lomé (Fig. 2B). The minimum number of larvae collected was 198 in a trapping site in the Adétikopé commune. Conversely, a total of 3063 *Ae. aegypti* larvae were collected in a single ovitrap at Amoutiévé over the study period (Supplementary Material Table 1).

**Fig. 3:**
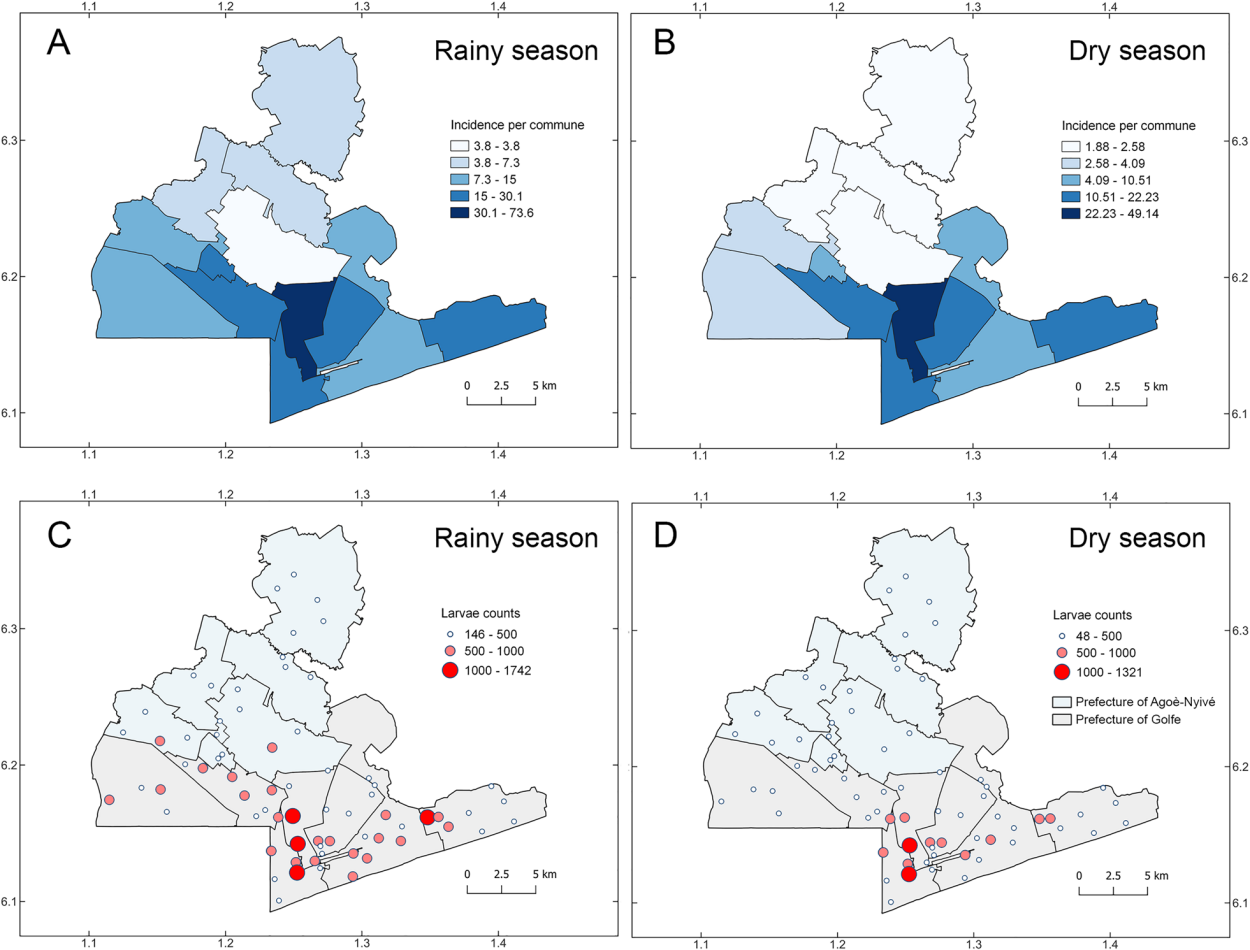
*Aedes aegypti* larval incidence per commune and counts across Grand Lomé, Togo, collected during the rainy and dry seasons. Eggs and larvae collected in 2022–2023 during the rainy (**A** and **C**) and dry seasons (**B** and **D**) were reared in the laboratory, and the incidence of *Ae. aegypti* larvae per commune was calculated using total numbers of larvae divided by total population for 2022 (**A** and **B**). Counts of *Ae. aegypti* larvae per ovitrap were categorized as low, medium, or high to highlight patterns of larval abundance across Lomé communes (**C** and **D**)

A total of 33,127 (62.78%) of the *Ae. aegypti* larvae were collected in Grand Lomé during the rainy season (Table 2). The communes of Bè-Ouest, Amoutiévé, and Aflao-Gakli had the largest larval incidence during this period, with 73.60, 30.08, and 23.73 per 1000 population, respectively (Table 2, Fig. 3A). Notably, these were also the communes driving the highest incidence over the entire study period (Figs. 2A and 3A). The lowest number of *Ae. aegypti* larvae collected in an individual trap during the rainy season was 146 at Adétikopé commune, and the highest number was 1742 larvae in a trap from Amoutiévé commune (Supplementary Material Table 1).

**Table 2:** Incidence of *Aedes aegypti* larvae during the rainy season in Grand Lomé

Communes	Commune capitals	*Ae. aegypti* larvae	Incidence of *Ae. aegypti* larvae
Agoè-Nyivé 1	Agoè-Nyivé	1190	3.75
Agoè-Nyivé 2	Légbassito	942	7.35
Agoè-Nyivé 3	Vakpossito	953	20.04
Agoè-Nyivé 4	Togblékopé	872	5.64
Agoè-Nyivé 5	Sanguéra	1311	10.47
Agoè-Nyivé 6	Adétikopé	692	6.27
Golfe 1	Bè-Est	5261	14.96
Golfe 2	Bè-Centre	2883	21.17
Golfe 3	Bè-Ouest	3884	73.60
Golfe 4	Amoutiévé	4688	30.08
Golfe 5	Aflao-Gakli	4034	23.73
Golfe 6	Baguida	4121	22.69
Golfe 7	Aflao-Sagbado	2296	8.90
Totals	Grand Lomé (total)	33,127	15.14

During 2022–2023, a total of 70 ovitraps were deployed across the 13 communes of Grand Lomé. The incidence of *Ae. aegypti* larvae was calculated using the human population of each commune in 2022 as denominator (see methods)

**Fig. 4:**
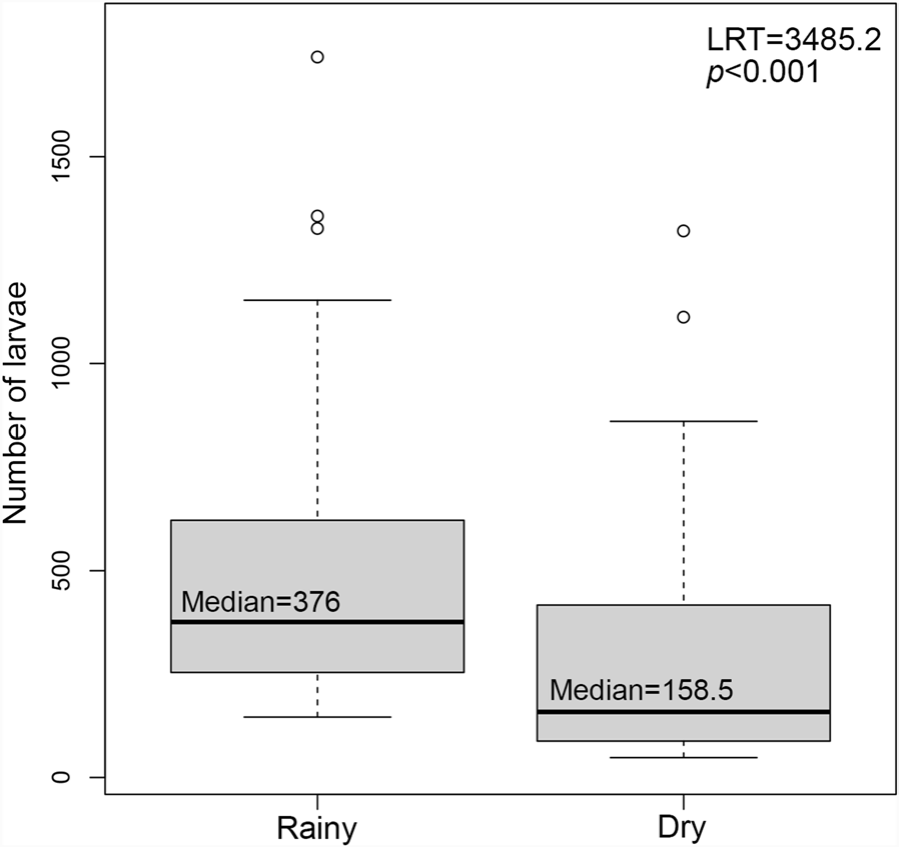
Comparison of *Aedes aegypti* larval counts between the rainy and dry seasons in Grand Lomé, Togo. We used a generalized linear mixed model to compare larval counts between Lomé seasons and found a statistically significant difference between larval abundance, with higher numbers in the rainy season (median = 376 *Ae. aegypti* larvae). LRT, likelihood ratio test

During the dry season, a total of 19,641 (37.22%) of the *Ae. aegypti* larvae were collected, which was roughly half the larvae collected during the rainy season (Tables 3 and 2, Fig. 3B). Bè-Ouest and Amoutiévé remained as the communes with the highest incidence during this period, with 49.14 and 22.23 larvae per 1000 population, respectively. The commune with the third-highest incidence in this period was Bè-Centre, with 14.26 larvae per 1000 population. All incidence values were lower than those of the rainy season (Tables 2 and 3, Fig. 3B). Communes including Adétikopé, Togblékopé, and Légbassito showed lower incidence overall (i.e., 2.58, 2.47, and 2.45 per 1000 population, respectively; Table 3). The maximum number of *Ae. aegypti* larvae collected in an individual trap was 1321 and the minimum was 48, which was lower than in the rainy season (Supplementary Material Table 1, Fig. 3C and 3D). A statistically significant difference was detected in the number of *Ae. aegypti* larvae collected between the rainy and dry seasons, with 1.69 times more larvae collected during the rainy season (Fig. 4, LRT = 3485.2, *P* < 0.001).

**Table 3:** Incidence of *Aedes aegypti* larvae during the dry season in Grand Lomé

Communes	Commune capitals	*Ae. aegypti* larvae	Incidence of *Ae. aegypti* larvae
Agoè-Nyivé 1	Agoè-Nyivé	598	1.88
Agoè-Nyivé 2	Légbassito	314	2.45
Agoè-Nyivé 3	Vakpossito	500	10.51
Agoè-Nyivé 4	Togblékopé	381	2.46
Agoè-Nyivé 5	Sanguéra	507	4.05
Agoè-Nyivé 6	Adétikopé	284	2.57
Golfe 1	Bè-Est	3264	9.28
Golfe 2	Bè-Centre	1941	14.26
Golfe 3	Bè-Ouest	2593	49.13
Golfe 4	Amoutiévé	3465	22.23
Golfe 5	Aflao-Gakli	2320	13.64
Golfe 6	Baguida	2420	13.32
Golfe 7	Aflao-Sagbado	1054	4.08
Totals	Grand Lomé (total)	19,641	8.98

During 2022–2023, a total of 70 ovitraps were deployed across the 13 communes of Grand Lomé. Incidence of *Ae. aegypti* larvae was calculated using the human population of each commune in 2022 as denominator (see methods)

**Fig. 5:**
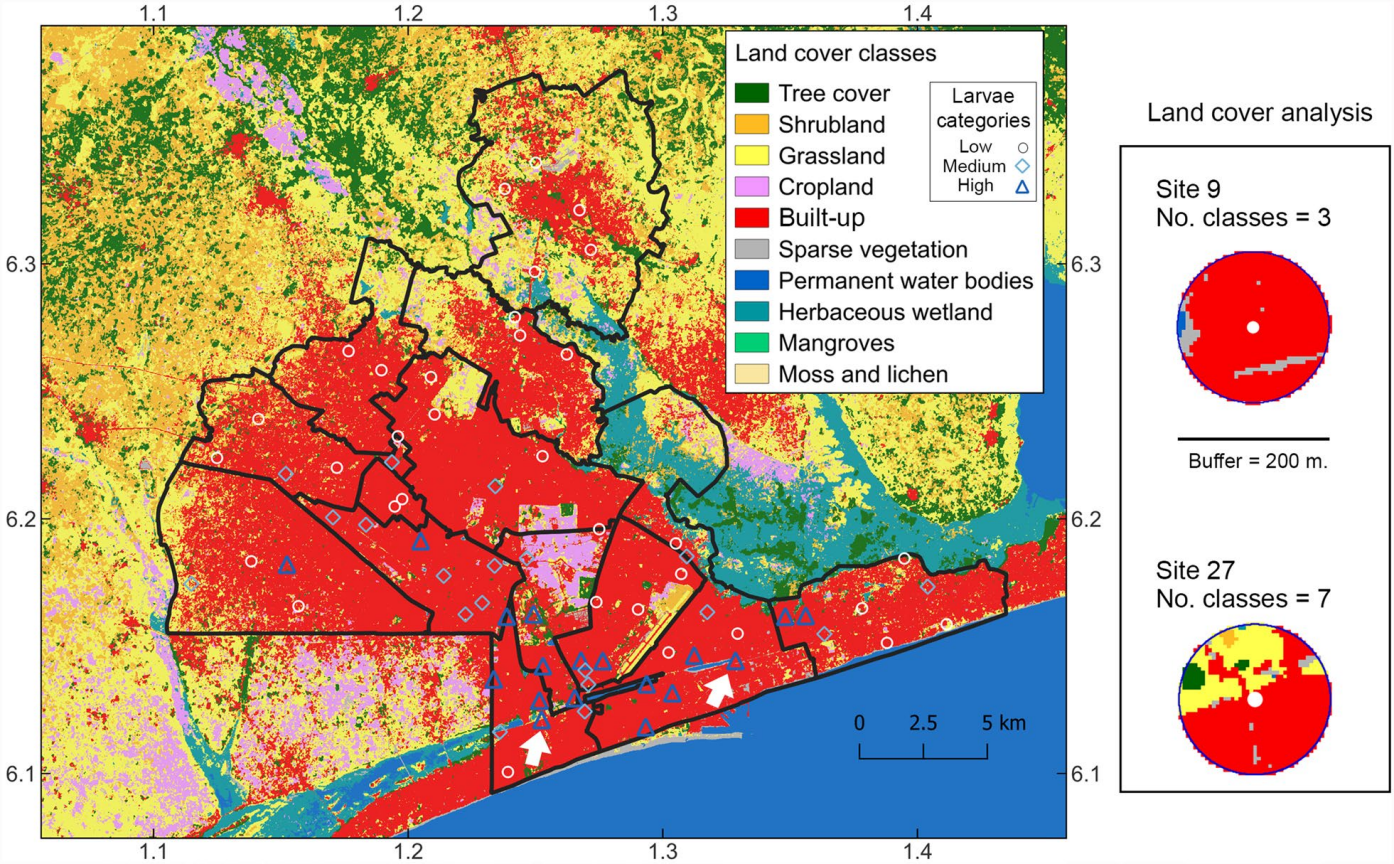
Land cover classes of Grand Lomé, Togo, according to the European Space Agency (ESA) WorldCover 10 m 2020 product. Eight classes of land cover are depicted in Grand Lomé. The category moss and lichen can be found in regions outside Grand Lomé. An example of land cover analysis units can be seen to the right based on sites 9 and 27 (white arrows at the south of Grand Lomé). A buffer of 200 m around the coordinates of trap collection allowed us to characterize the landscape surrounding the 70 traps of the study to make descriptive and statistical comparisons (see main text)

Categorizing the 70 ovitraps according to the number of larvae produced, 18 traps (25.71%) produced more than 1000 larvae (considered high), 19 traps (27.14%) 500–1000 larvae (i.e., medium), and 33 traps (47.14%) less than 500 larvae (i.e., low; Table 4). Eight land cover classes were represented by the ESA 10 m product (Table 4 and Fig. 5). The built-up category was the most common across Grand Lomé, followed by grassland and tree cover (Table 4 and Fig. 5). Visually, each buffer was composed mainly of pixels representing the built-up category and therefore suggesting a highly urban surface (Fig. 5). The Chi-square test assessing whether larval abundance categories and land cover classes were associated showed a significant relationship (*χ*^2^ = 2883.5, degrees of freedom = 14, *P* < 0.001). Consistently, tree cover, shrubland, and grassland had more pixels for traps with less than 500 larvae of *Ae. aegypti* (i.e., low category; Table 4). Land cover classes associated with the presence of water (i.e., permanent water bodies and herbaceous wetland) showed a larger number of land cover pixels for traps with medium and high numbers of *Ae. aegypti* larvae than for traps with lower numbers (Table 4).

**Table 4:** Distribution of land cover classes across ovitraps in Grand Lomé

Land cover ID	Land cover name	Total number of pixels available	Pixels available ovitraps class high (*n* = 18; 25.7%)	Pixels available at ovitraps class medium (*n* = 19; 27.1%)	Pixels available at ovitraps class medium+high (*n* = 37; 52.9%)	Pixels available at ovitrap class low (*n* = 33; 47.1%)
10	Tree cover	2992	377	321	698	1596
20	Shrubland	932	131	47	178	576
30	Grassland	7074	620	1235	1855	3364
40	Cropland	958	25	300	325	308
50	Built-up	122,303	21,201	21,964	43,165	35,973
60	Bare/sparse vegetation	656	142	71	213	230
80	Permanent water bodies	1554	539	238	777	NA
90	Herbaceous wetland	521	8	150	158	205

A buffer of 200 m was built around each of the 70 ovitraps deployed across Grand Lomé communes. Ovitraps were classified into three classes according to the number of *Ae. aegypti* larvae recovered: < 500 low, 500–1000 medium, and > 1000 high. We counted the number of pixels available per land cover category to determine the most common land cover type across different classes of ovitraps. Number and percentage of traps per class are depicted in the headers. Land cover ID: the name of the land cover category officially assigned in the ESA satellite product (see methods). NA, not applicable

Other species of mosquito larvae were collected during this study, including 902 *Anopheles* spp. larvae and 1272 *Culex* spp. larvae, with higher numbers found in the rainy season (Supplementary Material Table 2). No *Ae. albopictus* were found in our samples. The significance and epidemiological consequences of these other mosquito species will be reported separately.

## Discussion

This study identified *Ae. aegypti* larvae across the 13 communes of Grand Lomé, in both the rainy and the dry seasons. *Aedes albopictus* was not found in any of our samples despite the density of larvae collected. The presence of *Ae. aegypti* larvae in the capital of Togo throughout the year, with higher incidence during the rainy season (April–July), has important public health implications considering the plethora of pathogens this mosquito might carry, but especially considering the worldwide incidence of dengue [9].

In Lomé, a study conducted in 2017 at the Centre Hospitalier Universitaire (CHU) Sylvanus Olimpio in febrile patients showed that the prevalence of dengue was 17%, whereas that of malaria was 10.2% [20]. This research demonstrated that dengue virus was already circulating in Togo before the official public report of eight dengue cases in 2023 [12]. Indeed, dengue is poorly recognized by health professionals in the country [21], leading to underdiagnosis of dengue fever. Thus, by demonstrating the circulation of *Ae. aegypti* in Grand Lomé over the year, our study should alert public health authorities about the risk of an imminent dengue outbreak or outbreaks of other *Aedes*-borne febrile illnesses such as Zika, chikungunya, yellow fever, etc., that might be included in the differential diagnoses of unknown fevers in Togo.

The larger numbers of *Ae. aegypti* larvae collected during the rainy season indicate a seasonal peak of mosquito larval productivity in Grand Lomé. The incidence of *Ae. aegypti* larvae was more homogeneous across communes in the dry season than in the rainy season (Fig. 3A vs. B), which might be driven by anthropogenic activities, mosquitoes exploiting human-made container habitats, dry season intensity, and availability of humans as blood meal hosts [34, 35]. Human population and precipitation can influence the abundance of dengue virus vectors [36, 37], and *Ae. aegypti* anthropophilic behavior and association with urban areas have been documented historically [38, 39]. In fact, the expansion of urbanization is often associated with the emergence and spread of vector-borne diseases, creating favorable conditions for the survival of *Aedes* species and the spread of dengue, chikungunya, and Zika viruses [40]. We suggest that the high number of larvae obtained during the rainy season might be a consequence of two processes: (1) the resistance of *Ae. aegypti* eggs to desiccation during the dry season [41] and (2) the multiplication of breeding habitats during rains [18]. Our results corroborate those reported by Edig and colleagues [42], who showed that *Ae. aegypti* abundance peaks at the beginning of the rainy season and is generally positively correlated with rainfall in West Africa, and results reported by Noleto and colleagues, who observed a sudden increase in larval numbers of *Aedes* spp. in northeastern Brazil during the rainy season [43]. These specific adaptive characteristics may have favored the species’ worldwide distribution [44].

The distribution and abundance of mosquitoes are linked to the breeding sites that are favorable to their reproduction [18]. The presence of *Ae. aegypti* larvae in all of the communes of Grand Lomé suggests an entomological risk of arbovirus transmission, including a dengue outbreak or the re-emergence of yellow fever [45, 46].

Nearly half of the traps (*n* = 33) recovered less than 500 larvae. A total of 19 traps collected between 500 and 1000 larvae, and 18 traps collected more than 1000 larvae (Table 4). Some patterns were appreciated when combining traps from the medium and high larval abundance categories (Table 4). Specifically, pixels for the built-up category representing urban environments were present in higher numbers for traps capturing more than 500 *Ae. aegypti* larvae. Nevertheless, for traps capturing less than 500 *Ae. aegypti* larvae (the low abundance category), the built-up pixel category also represented most of the land area available (Fig. 5). This highlights how well-adapted *Ae. aegypti* is to urban environments, and previous work has demonstrated how poorly planned rapid urbanization facilitates the spread of arboviruses [47]. In fact, the expansion of urbanization is often associated with the emergence and spread of vector-borne diseases by creating favorable conditions for the survival of *Aedes* species and the spread of dengue, chikungunya, or Zika viruses [40]. This fact has been highlighted in several western African countries, particularly Abidjan in the Ivory Coast [15, 42] and other metropolitan regions such as Córdoba city in Argentina, Tucson in the US state of Arizona, and northern Thailand [32, 48, 49].

The presence of a high number of pixels related to the presence of water bodies for traps in the medium and large category of larval abundance (Table 4) can be related to a permanent source for mosquito development throughout the year (Fig. 5), including during the dry season (Fig. 4). Also, it is worth noting the presence of land cover categories related to vegetation cover and the presence of traps with lower numbers of *Ae. aegypti* larvae (Table 4, Fig. 5). Conversely, in other contexts, studies have shown that traps in moderate vegetation cover could collect more *Aedes* mosquitoes [50]. This could be associated with highly exophilic and anthropophilic mosquito behavior, preferring human hosts over other mammals [35, 51], and therefore highlighting the importance of preserving areas with vegetation [46]. According to Benitez et al. [32], areas with the lowest tree vegetation and highest building construction could be high-risk areas for dengue virus transmission.

As one limitation of the present study, we used tap water instead of rain for the ovitraps. It may be that chemicals in tap water caused female *Ae. aegypti* to actively avoid collection sites, preventing a larger recovery of larvae. However, the collection of larvae across all ovitraps, in all the communes, and during the rainy and dry seasons suggests that underestimation is unlikely. More studies are needed to address this particularity.

In this study, larvae and mosquitoes were identified only by morphological and not molecular methods. Although the latter might be recommended, the experience from entomological studies in our laboratory is enough to confidently recognize the species mentioned here. Systematic deployment of ovitraps across communes would have been preferred in terms of interpretation of larval abundance and the calculation of other entomological parameters such as larvae and mosquito density or richness and diversity patterns. However, our aim was to assess whether *Ae. aegypti* was present or absent in different seasons across Grand Lomé communes, which we effectively determined with our current sampling. With this set of results, next steps include the design of a systematic sampling to disentangle further biological and entomological questions related to *Ae. aegypti* biology in Togo and their ability to promote potential dengue outbreaks.

The incidence of *Ae. aegypti* larvae calculated in this study assumes a direct relationship between this statistic and the potential risk of dengue detectability at the commune level in Grand Lomé. Theoretically, outbreaks of dengue in less-human-populated communes with a larger number of larvae—i.e., larger incidence—will have a larger number of female mosquitoes ready to bite and transmit arbovirus, and therefore might be easier to detect. This hypothesis, although valid, should be explored accordingly in future attempts to clarify dengue outbreak risk in Grand Lomé.

It is important to mention that the utilization of the ESA 10 m satellite product for land cover analysis might be suboptimal in terms of geographical resolution. Specifically, higher-resolution images (i.e., 1–2 m resolution) could be more informative for differentiating between land cover classes. However, our approach leverages the readiness of Google Earth Engine tools for assessing ecological questions of *Ae. aegypti* in Lomé depicting overall characteristics driving larval density patterns. Further studies should refine our analysis by deriving land cover products beyond 10 m resolution.

Finally, in our study, we leveraged the ovitrap setting based on convenience and probability of ovitrap recovery (see methods). In the future, studies looking for specific *Aedes* parameters should determine the number of ovitraps placed per commune as a function of the human population to better understand larval incidence.

## Conclusions

This study offers an overview of the distribution of *Ae. aegypti* in the health region of Grand Lomé in Togo. The presence of *Ae. aegypti* larvae at all collection sites and their increased abundance during the rainy season represent a potential risk of arbovirus transmission, especially considering the recent reports of dengue cases in Togo. Larval incidence was higher in the communes of Golfe than in those of Agoè-Nyivé, and therefore largely concentrated in the southern part of the study area irrespective of the season. Further seasonal surveillance to understand the types of larval habitats will allow vector control programs to target those areas capturing larger larval numbers. Vector control measures, such as larval source management and adult mosquito control to be developed, are urgently needed to prevent the spread of arboviruses in the country. Moreover, larger surveillance of arboviruses might discover the current underdiagnosis of dengue fever or other diseases in Togo.

## Supplementary Information

Supplementary Material 1: The supplementary material includes three tables. Table 1. Counts of *Aedes aegypti *larvae obtained by trap and number of reared adults. Table 2. Non-*Aedes aegypti* larvae identified in the collection. Table 3: Number of pixels per category of land cover data for each ovitrap and each commune involved in the study.

## Data Availability

The data supporting the findings of the study must be available within the article and/or its supplementary materials, or deposited in a publicly available database.
